# Does laparoscopic management of deep infiltrating endometriosis improve quality of life? A prospective study

**DOI:** 10.1186/1477-7525-9-98

**Published:** 2011-11-06

**Authors:** Mohamed Mabrouk, Giulia Montanari, Manuela Guerrini, Gioia Villa, Serena Solfrini, Claudia Vicenzi, Giuseppe Mignemi, Letizia Zannoni, Clarissa Frasca, Nadine Di Donato, Chiara Facchini, Simona Del Forno, Elisa Geraci, Giulia Ferrini, Diego Raimondo, Stefania Alvisi, Renato Seracchioli

**Affiliations:** 1 Minimally Invasive Gynaecological Surgery Unit, S.Orsola Hospital, University of Bologna, Italy; 2 Department of Obstetrics and Gynecology, Alexandria University, Egypt

## Abstract

**Background:**

Deep infiltrating endometriosis (DIE) can affect importantly patients' quality of life (QOL). The aim of this study is to evaluate the impact of the laparoscopic management of DIE on QOL after six months from treatment.

**Methods:**

It is a prospective cohort study. In a tertiary care university hospital, between April 2008 and December 2009, 100 patients underwent laparoscopic management of DIE and completed preoperatively and 6-months postoperatively a QOL questionnaire, the short form 36 (SF-36).

Quality of life was measured through the SF-36 scores. Intra-operative details of disease site, number of lesions, type of intervention, period of hospital stay and peri-operative complications were noted.

**Results:**

Six months postoperatively all the women had a significant improvement in every scale of the SF-36 (p < 0,0005). Among patients with intestinal DIE, significant differences in postoperative scores of SF-36 were not detected between patients submitted to nodule shaving and segmental resection (p > 0.05). There was no significant difference in the SF-36 scores at 6 months from surgery between patients who received postoperative medical treatment and patients who did not (p > 0.05).

**Conclusions:**

Laparoscopic excision of DIE lesions significantly improves general health and psycho-emotional status at six months from surgery without differences between patients submitted to intestinal segmental resection or intestinal nodule shaving.

## Background

Deep infiltrating endometriosis (DIE) defined as the infiltration of anatomic structures, pelvic organs, or both, is a source of pelvic pain and altered quality of life [1-4]. The exact incidence of DIE in the general population is not known, but it is estimated to affect 20% of women with endometriosis [5].

Although many studies demonstrated that surgical resection of all endometriotic lesions is recommended to relieve pain, its effectiveness is still debated [5-16]. In addition, the risk of serious complications inherent to this type of surgery has been estimated between 4 and 6% of cases [17,18] with a high rate of de novo neurological disorders [19]. It has been demonstrated that the secondary effects of surgical treatment and the persistence of some symptoms can have an impact on the patient's quality of life [20]. Furthermore, when we treat endometriosis we have to consider that it is a benign disease which affects young, professionally active women, who may plan to conceive.

In our opinion, quality of life (QOL) evaluation is important to assess the overall effects of radical excision of DIE, taking in consideration that endometriosis is a pathology that has symptoms which may disrupt working ability, social relationships and sexual functioning.

Several general questionnaires have been recommended for QOL assessment ([2,3,20-23]). Between them, the short form 36 (SF-36) has been used to evaluate the improvement in QOL in patients submitted to laparoscopic surgery [4,24] for endometriosis and, in general, to evaluate the impact of endometriosis and its treatment on women's health-related quality of life [25].

Two surgical approaches are usually employed in management of deep endometriosis with intestinal muscularis involvement: segmental resection and nodule excision. This latter approach may be performed without opening the intestinal lumen (shaving) or by removing the nodule along with the surrounding intestinal wall (full thickness or disc excision). A strong debate continues between advocates of the nodule excision techniques and supporters of segmental resection. To date, there is no consensus made about the surgical management of deep intestinal endometriosis [26]. Recently SF-36 has been proposed as a complementary tool to select and inform women who might benefit from laparoscopic segmental resection for endometriosis [27].

In the present study we sought to prospectively evaluate the impact of laparoscopic management of DIE on the patients' QOL. We also aimed to investigate whether or not a greater level of QOL improvement can be achieved by performing segmental resection rather than nodule excision in patients with deep intestinal endometriosis.

## Methods

Full ethical approval was obtained from the local ethics committee to the study protocol (155/2008U/Oss).

### Protocol and surgical treatment

From April 2008 through December 2009, in the Minimally Invasive Gynaecological Surgery Unit, S. Orsola-Malpighi Hospital, University of Bologna, a consecutive series of 120 patients with preoperative diagnosis of deep infiltrating endometriosis agreed to take part to the study protocol.

Exclusion criteria were as follows: major medical conditions, psychiatric disorders, current or past (within 6 months from study enrolment) use of drugs affecting cognition, vigilance and/or mood.

For each patient, general data were assessed together with history of surgical treatment for endometriosis and the scoring of pelvic pain symptoms using a 10-point visual analogue scale (VAS).

All women underwent gynaecological examination, pelvic trans-vaginal and abdominal ultra-sonography in order to evaluate the presence of pelvic endometriosis before surgery. Other diagnostic tests were performed when indicated, as previously described [28,29].

All women were scheduled for laparoscopic management of deep infiltrating endometriosis and they gave informed written consent to surgical treatment and the possible use of their anonymous data for research purposes. The surgical strategy was complete laparoscopic excision of all visually suspected endometriotic lesions and the laparoscopic procedures were performed by the same surgeon (R.S.). The surgical team had a consistent background in laparoscopic treatment of patients with DIE. Laparoscopic resection of endometriosis was performed as previously described [28-32]. In particular, women were scheduled for segmental recto-sigmoid resection when bowel function was greatly impaired and when radiological diagnosis of intestinal endometriosis confirmed the presence of intestinal lesions associated with marked restriction of the bowel lumen. Moreover, deciding the necessity of intestinal resection or intestinal nodule shaving, we took into account endometriosis and intestinal symptoms, impairment of quality of life due to intestinal symptoms, desire of pregnancy and finally the intra-operative evaluation performed by the gynaecological surgeon and the general surgeon. Only after histological confirmation of diagnosis, the patients were asked to continue the postoperative phase of the study. Deep infiltrating endometriosis (DIE) was considered histologically confirmed when the lesion penetrates >5 mm under the peritoneal surface [33]. We considered intestinal DIE when the lesion infiltrated the muscularis [34].

After surgical treatment patients were recommended to use medical therapy to prevent anatomical lesion recurrences and symptoms relapse. All patients were asked to undergo a follow-up visit six months after surgery. During the follow-up visit, patients underwent physical examination and trans-vaginal ultrasonography to evaluate symptoms and/or anatomical relapse of endometriotic nodules. Women were asked to complete the SF-36 Questionnaire and to rank their symptom intensity using the same numerically rated VAS used preoperatively.

### QOL assessment

The SF-36 is a multi-purpose health survey with 36 questions. It yields an eight-scale profile of functional health and well-being scores, as well as psychometrically based physical and mental health summary measures (standardized). The eight scales are hypothesized to form two distinct higher-ordered clusters due to the physical and mental health variance that they have in common. Among the eight scales, three [physical functioning (PF), role physical (RP), bodily pain (BP)] correlate most strongly with the physical component and contribute most to the Physical Component Summary (PCS) score. The mental component correlates best with the mental health (MH), role emotional (RE) and social functioning (SF) scores, which also contribute most to the Mental Component Summary (MCS) score. Two of the scales [vitality (VT) and general health (GH)] have noteworthy correlations with both components. All the women completed preoperatively and 6-months postoperatively the SF- 36 questionnaire, Italian version, release 1.6 [35].

### Statistical Analysis

All continuous variables were expressed in terms of mean ± standard deviation of the mean. The Kolmogorov Smirnov test was performed to assess the normal distribution. The Paired t test was performed to assess the difference between score means when the data were normally distributed; otherwise the Wilcoxon Test was used to check T test results. One Way ANOVA was performed to assess the difference of the score means between patients with and without the studied characteristic. When the Levene test for homogeneity of variances was significant (p < 0.05) the Mann Whitney test was used to check ANOVA results. Pearson's Chi square test, calculated by Exact Method, was performed to investigate the relationships between grouping variables.

Pearson's correlation analysis was used to test relationship between continuous variables. For all tests p < 0.05 was considered significant. Statistical Analysis was performed by means of the Statistical Package for the Social Sciences (SPSS) software version 15.0 (SPSS Inc., Chicago, USA).

## Results

Of the 120 patients assessed for eligibility, 20 were excluded. Seven did not have a histologically confirmed DIE following laparoscopic excision of their disease. Nine did not complete the questionnaire. Four did not come to the 6 months follow-up visit. Consequently, 100 patients were enrolled in our study. Average age at the time of surgery was 34.2 ± 4 years (range [23-39]) and mean body mass index was 21.6 ± 2.7 Kg/m² (range [19-32]). Regarding previous surgical treatments for endometriosis, 27% (27/100) had one previous procedure, 4% (4/100) had two and one patient had three previous interventions. Operative findings, surgical procedures, additional procedures performed and complications are summarized in Table 1.

**Table 1:** Surgical procedures, additional surgical procedures, intra-operative and postoperative complications of the laparoscopic management of DIE

	Number
**Surgical procedures:**	
- Recto-vaginal septum nodule resection	62
- Intestinal nodule shaving	50
- Segmental intestinal resection	16
- Vagina nodule resection	32
- Utero-sacral ligaments nodule resection	44
- Bladder nodule resection	41
- Ureteral nodule resection:	18
- Ureterolyisis	15
- Segmental ureteral resection with end to end anastomosis	3

**Additional surgical procedures performed:**	
- Appendectomy	4
- Nephrectomy	1
- Temporary colostomy	1

**Intra-operative complications**	
- Bowel injury	0
- Bladder injury	0
- Ureteral injury	0
- Vascular injury	1
- Blood loss exceeding 500 ml - Conversion to laparotomy	1 0

**Postoperative complications**	
- Transient fever > 38 °C	8
- Transient urinary retention	3
- Urinary incontinence	1
- Uretero-vaginal fistula	1
- Recto-vaginal fistula	1

### SF 36 Scores

After laparoscopic surgery for DIE, at 6-months follow up, a significant improvement was observed in the SF-36 total score, in the SF-36 component summaries and in every scale of the SF-36 (p < 0.0005) (Table 2).

**Table 2:** Mean (± Standard deviation) preoperative and postoperative scores of the scale of SF-36

	BEFORE	AT 6 MONTHS FOLLOW-UP	P value
**SF-36 total score**	**49 ± 20**	**71 ± 17**	**< 0.0005**

Physical Component Summary	49 ± 19	70 ± 17	< 0.0005

Physical Function	77 ± 23	90 ± 14	< 0.0005
Role - Physical	40 ± 39	77 ± 35	< 0.0005
Body pain	38 ± 20	68 ± 24	< 0.0005

Mental Component Summary	47 ± 20	66 ± 17	< 0.0005

Social Functioning	50 ± 22	72 ± 22	< 0.0005
Role Emotional	40 ± 40	76 ± 33	< 0.0005
Mental Health	54 ± 18	65 ± 16	< 0.0005

General Health	47 ± 21	59 ± 19	< 0.0005

Vitality	46 ± 19	57 ± 17	< 0.0005

Among patients with intestinal DIE, significant differences in postoperative scores of SF-36 were not detected between patients submitted to intestinal nodule shaving and segmental intestinal resection (p > 0.05) (Table 3).

**Table 3:** Mean improvement (± Standard deviation) of SF-36 scores six months after surgery.

	INTESTINAL RESECTION (16 patients)	NODULE EXCISION (50 patients)	P value
**ΔSF-36 total score**	**37 ± 36**	**35 ± 42**	**0.08**

ΔPhysical Component Summary	36 ± 35	35 ± 41	0.23

ΔPhysical Function	14 ± 25	13 ± 24	0.30
ΔRole - Physical	41 ± 46	43 ± 40	0.06
ΔBody pain	32 ± 31	30 ± 26	0.41

ΔMental Component Summary	24 ± 42	26 ± 36	0.09

ΔSocial Functioning	21 ± 32	21 ± 26	0.08
ΔRole Emotional	35 ± 51	38 ± 41	0.07
ΔMental Health	8 ± 24	10 ± 19	0.09

ΔGeneral Health	10 ± 22	11 ± 20	0.06

ΔVitality	10 ± 22	11 ± 18	0.07

Comparison between patients submitted to segmental intestinal resection and patients submitted to intestinal nodule excision.

Pain scores were significantly improved after six months from surgical treatment (p < 0.05). Preoperatively 99% of women had dysmenorrhea (mean VAS score of 7 ± 3), 76% dyspareunia (mean VAS score of 5 ± 3), 63% chronic pelvic pain (mean VAS score of 4 ± 3), 67% dyschezia (mean VAS score 5 ± 4) and 34% had dysuria (mean VAS score of 2 ± 3). Postoperatively, at 6 months follow up, 23% of women reported dysmenorrhea (mean VAS score 1 ± 3), 23% dyspareunia (mean VAS score of 1 ± 2), 18% chronic pelvic pain (mean VAS score of 1 ± 2), 17% dyschezia (mean VAS score of 1 ± 2) and 6% dysuria (mean VAS score of 0 ± 1).

On pelvic examination and through ultrasound exam, there were no cases of anatomical recurrence at 6-months follow-up.

Seventy-one percent of patients (71/100) assumed postoperative hormonal treatment (33 with cyclic, 27 with continous oral estro-progestogenic; 4 with cyclic estro-progestogenic, 2 with continous vaginal ring; 3 with oral progestins and 2 with estro-progestogenic cyclic patch). There were no significant difference in the SF-36 postoperative scores between patients who received postoperative medical treatment and patients who did not (p > 0.05). outcomes after surgical treatment of DIE.

## Discussion

By performing this trial and reviewing the available literature, we tried to answer some questions related to this particular pathology, DIE:

### 1) Is it important to consider objective QOL evaluation in patients with DIE?

In 2000, Garry et al. affirmed that endometriosis exerts a profoundly adverse effect on the personal life and relationships of patients [2]. The intensity and frequency of symptoms, their association and concomitant infertility, the secondary effects of medical and surgical management, symptoms persistence after treatment, disease relapse and the need of continuing a therapy for a long term affect negatively quality of life [20]. We believe that one of the primary goals of the management of endometriosis is not only symptom reduction, but also improvement of the overall patient's quality of life. In this perspective, the evaluation of the efficacy of surgical management of endometriosis only in terms of pain and symptoms improvement seems insufficient. Recently Dubernard et al. proposed SF-36 questionnaire as a tool that can predict the degree of change in QOL after laparoscopic management of posterior DIE [27], delineating a new approach of DIE in which QOL evaluation can guide the management of the disease.

### 2) Does laparoscopic management of DIE improve QOL?

After laparoscopic surgery for DIE, at six-month follow up, we observed a significant improvement in all scales of the SF-36.

Many studies confirmed that laparoscopic treatment of endometriosis is effective in relieving dysmenorrhoea, dyspareunia, non-menstrual pelvic pain and dyschezia ([2,33,34,36]).

In a randomized placebo-controlled trial of 39 women, Abbott et al. demonstrated that laparoscopic excision of endometriosis is more effective than placebo on pain reduction and quality of life improvement at 12 months from surgery [21]. However, in this trial, authors evaluated all rAFS stages of endometriosis and not DIE.

Jones et al. included in their study on laparoscopic ablative surgery for endometriosis, the evaluation not only of pain scores, but also of patient satisfaction scores. They showed that women with rAFS stage III-IV of endometriosis who underwent treatment presented a high rate (87.7%) of satisfaction [36].

In 2000, Garry et al showed that radical laparoscopic excision of endometriosis stage III and IV of rAFS significantly improved the physical component score of the QOL questionnaire, returning the score value to a normal range. The mental component score improved too, but this was not statistically significant and failed to reach a normal range four months after treatment [2]. This study analyzed prospectively 57 patients and was performed using Short Form 12 (SF12) and Euro QOL (EQ-5D) questionnaire preoperatively and 4 months after surgery. However, SF-12 questionnaire reproduces the eight scale profile with fewer levels than SF-36 scales and yields less precise scores [27].

In 2003, Abbott et al. studied 176 women who underwent laparoscopic excision of endometriosis, evaluating long term outcome through the use of QOL questionnaire [3]. The results evidenced that women with endometriosis have an impaired QOL which improve after treatment in a significant manner. The increase in the physical component appeared greater than the mental component of the score. However the results of this prospective study with an evaluation of the QOL in the long-term may be affected by the high rate of women who did not respond to the follow-up questionnaire (26%).

### 3) Is there a difference in QOL improvement between patients who undergo nodule shaving or segmental intestinal resection?

We found that there was no significant difference in the six-month postoperative improvement of SF-36 scores among women with intestinal DIE who underwent nodule shaving or segmental intestinal resection.

In the literature the debate regarding the surgical management of intestinal DIE is current [37]. While some studies evidenced a significant QOL improvement in women treated with colorectal segmental resection [38-41], others suggested nodule excision or shaving, as a first choice procedure. These authors retrieved an increased risk of postoperative complications together with de novo intestinal and urological symptoms appearance in patients submitted to segmental intestinal resection [37,40,42-44]). Recently Roman et al. in a retrospective study evidenced that women undergoing colorectal resection when compared with women managed by nodule excision, were more likely to present several unpleasant functional digestive outcomes and urinary dysfunctions [37]. However, the choice of colorectal resection is supported by the fact that the absence of bowel resection in women with DIE and intestinal endometriosis is the factor most strongly associated with recurrence rate [8]. Moreover, there are studies which showed that microscopic endometriotic lesions usually exist around the main rectal nodule [15,45]. In our opinion, important issues to be considered, when deciding the need and the type of surgery in women with intestinal endometriosis, are the actual status and the expected improvement of the patient's QOL, as well as the potential functional outcomes of surgery. Finally, further prospective randomized studies are necessary to assess which surgical management is more indicated in patients with intestinal DIE.

### 4) Does postoperative hormonal treatment influence QOL at six-month follow up?

We did not find any significant difference in all SF-36 scores between patients submitted to the surgical treatment alone and patients who received six-month postoperative hormonal treatment.

Considering DIE, it has been shown that continuous post-operative hormonal treatment might prevent pain recurrences after surgical removal of deep infiltrating nodules [46]

Regarding the elective postoperative management of endometriosis, data from the randomized trials are controversial in terms of pain recurrence and anatomical relapse. A Cochrane review of 2004 showed that post-surgical hormonal suppression of endometriosis compared to surgery alone (either no medical therapy or placebo) showed no benefit for the outcomes of pain [47]. Muzii et al [48] found no significantly difference in the recurrence rates of pain at follow-up between patients receiving oral contraceptives pills (9.1%) and untreated patients (17.1%). Furthermore, Koga et al. found in their retrospective study that a mean postoperative treatment of 9.5 months did not influence recurrence [49]. Recently, different studies evidenced an important role of long term postoperative use of oral contraceptive on symptoms and disease recurrence [50-52].It seems that the length of the treatment is, therefore, an important factor in the long-term efficacy of therapy. However, all these trials considered only pain recurrence and anatomical relapse, ignoring QOL evaluation. Further trials are necessary to assess whether postoperative medical therapy impact on the QOL. Recently, some authors adopted the concept that in the treatment of DIE, it is most likely that medical and surgical treatments should be associated [26,53].

Certain limitations of this study must be underlined. Our results may be influenced by the fact that one third of the women (31%) involved in the study had previously been surgically treated for endometriosis. In these women, the previous failed surgery may bias the QOL perception with lower preoperative SF-36 scores. Second, more than an half of patients (56%) were taking hormonal therapy before surgical treatment and a large proportion (71%) of women was given postoperative hormonal treatment. This may potentially have a significant bias on the symptoms and QOL perception of these women. However, as it has been stated by recent studies, long term outcomes of the surgical treatment of endometriosis are positively correlated with the assumption of postoperative medical therapy leading to the conclusion that only the combination of surgery plus medical therapy may guarantee long term effect [53]. Third, our study evaluated QOL after only six months postoperatively, which seems to be a short time to complete the recovery from this complex surgery. However, there is an ongoing study in our centre aiming to assess long term QOL outcomes after surgical treatment of DIE.

## Conclusion

We found that laparoscopic excision of DIE lesions appreciably improves general health and psycho-emotional status at six-month follow up without differences between patients submitted to intestinal segmental resection or nodule shaving. We strongly believe that objective QOL assessment should be considered as a complementary index to evaluate need and success of therapeutic interventions in DIE.

## List of abbreviations

The abbreviations used in the manuscript are summarized: BP: bodily pain; DIE: deep infiltrating endometriosis; GH: general health; MCS: mental component summary; MH: mental health; PCS: physical component summary; PF: physical functioning; RE: role emotional; RP: role physical; QOL: quality of life; SF: social functioning; SF-36: short form 36; VAS: visual analogue scale; VT: vitality.

## Competing interests

The authors declare that they have no competing interests.

## Authors' contributions

All authors read and approved the final manuscript. They contributed to the manuscript as follows: GM, MM and SR were involved in the conception and design of this study, in the analysis and interpretation of data, and in development and review of the manuscript for intellectual content. MG and GM were involved in the analysis and interpretation of data and in development and review of the manuscript for intellectual content. VG, MG and VC were involved in the interpretation of data and in review of the manuscript for intellectual content.

FC, SA, DDN and FC were involved in the collection of data. FG, DFS, GE, SS and RD were involved in the statistical analysis. ZL was involved in the manuscript revision.
